# A Low-Gluten Diet Reduces the Abundance of Potentially Beneficial Bacteria in Healthy Adult Gut Microbiota

**DOI:** 10.3390/nu17152389

**Published:** 2025-07-22

**Authors:** Eve Delmas, Rea Bingula, Christophe Del’homme, Nathalie Meunier, Aurélie Caille, Noëlle Lyon-Belgy, Ruddy Richard, Maria Gloria Do Couto, Yohann Wittrant, Annick Bernalier-Donadille

**Affiliations:** 1 UMR454 MEDIS, Université Clermont Auvergne/INRAE, 63000 Clermont-Ferrand, France; eve.delmas@inrae.fr (E.D.); christophe.del-homme@inrae.fr (C.D.); maria-gloria.do-couto@inrae.fr (M.G.D.C.); 2 UMR 1019 Nutrition Humaine, Université Clermont Auvergne/INRAE, 63000 Clermont-Ferrand, France; rea.bingula@uca.fr (R.B.); yohann.wittrant@inrae.fr (Y.W.); 3 CRNH Auvergne, CHU Clermont-Ferrand, 63000 Clermont-Ferrand, France; nathalie.meunier@uca.fr (N.M.); acaille@chu-clermontferrand.fr (A.C.); nlyonbelgy@chu-clermontferrand.fr (N.L.-B.); ruddy.richard@uca.fr (R.R.)

**Keywords:** healthy adults, low gluten diet, gut microbiota, microbial metabolism

## Abstract

**Background/Objectives:** An increasing number of apparently healthy individuals are adhering to a gluten-free lifestyle without any underlying medical indications, although the evidence for the health benefits in these individuals remains unclear. Although it has already been shown that a low- or gluten-free diet alters the gut microbiota, few studies have examined the effects of this diet on healthy subjects. Therefore, our aim was to evaluate whether and how a prolonged low-gluten diet impacts gut microbiota composition and function in healthy adults, bearing in mind its intimate link to the host’s health. **Methods:** Forty healthy volunteers habitually consuming a gluten-containing diet (HGD, high-gluten diet) were included in a randomised control trial consisting of two successive 8-week dietary intervention periods on a low-gluten diet (LGD). After each 8-week period, gut microbiota composition was assessed by 16S rRNA gene sequencing, molecular quantification by qPCR, and a cultural approach, while its metabolic capacity was evaluated through measuring faecal fermentative metabolites by ^1^H NMR. **Results:** A prolonged period of LGD for 16 weeks reduced gut microbiota richness and decreased the relative abundance of bacterial species with previously reported potential health benefits such as *Akkermansia muciniphila* and *Bifidobacterium* sp. A decrease in certain plant cell wall polysaccharide-degrading species was also observed. While there was no major modification affecting the main short-chain fatty acid profiles, the concentration of the intermediate metabolite, ethanol, was increased in faecal samples. **Conclusions:** A 16-week LGD significantly altered both composition and metabolic production of the gut microbiota in healthy individuals, towards a more dysbiotic profile previously linked to adverse effects on the host’s health. Therefore, the evaluation of longer-term LDG would consolidate these results and enable a more in-depth examination of its impact on the host’s physiology, immunity, and metabolism.

## 1. Introduction

Gluten is a major dietary component of wheat, rye, and barley, and consists of a protein complex including large peptides such as gliadins and glutenins. These large peptides are resistant to proteolytic digestion and are barely broken down by human intestinal proteases. They escape digestion in the stomach and reach the small intestine where they can interact with the immune system, affect the intestinal permeability, and subsequently induce changes within the gut microbiota [1]. Gluten is associated with some diseases such as celiac disease, gluten ataxia, or non-coeliac gluten sensitivity. In patients with celiac disease, an autoimmune disorder that develops in genetically susceptible individuals (about 1% of the European population), a gluten-free diet is prescribed as a lifelong treatment, in the absence of any medication. Eviction of gluten may also be recommended for other gluten-related disorders, such as non-celiac gluten/wheat sensitivity which causes a range of digestive symptoms (altered transit and/or abdominal pain) [2].

Over the past decade, there has been a significant increase in the adoption of a gluten-free lifestyle by healthy individuals, without any medical indication. These individuals have restricted gluten from their diet for a variety of reasons, including self-reported improved well-being, particularly digestive comfort, weight management, or the perception that gluten is harmful [3]. However, the evidence for the health benefits of a low-gluten diet in these healthy individuals remains critical as robust and convincing results are still lacking. Gluten avoidance may also have adverse effects in people with no evidence of gluten-related disease [4]. In the long term, a gluten-free diet was indeed shown to lead to dietary imbalances in celiac disease patients, characterised by low complex carbohydrate and fibre intakes, high fat and sugar intakes and micronutrient deficiencies (iron, calcium, magnesium, polyphenols, and vitamins D, E, and B) [5]. After one year on a gluten-free diet, patients with celiac disease were reported to be at a higher risk of developing metabolic syndrome [6]. This can be partly explained by higher fat and sugar contents as well as the higher glycaemic index of many gluten-free foods compared to their gluten-containing equivalents. The nutritional imbalances observed therefore call for long-term follow-up of celiac patients on a gluten-free diet, in order to promote nutritional education to achieve a balanced gluten-free diet and avoid the risk of developing metabolic syndrome.

Diet is also one of the major factors influencing the gut microbiota and plays a key role in host health. The gut microbiota is in a symbiotic relationship with the host, managing key functions such as energy production, maturation, and maintenance of the immune system or gut integrity [2]. Any change in the gut ecosystem can therefore affect the health of the host. In this context, a gluten-free diet, that is also characterised by a change in the type and content of fibre and a possible increase in fat and sugar [5], will directly affect the gut microbiota.

Changes in gut microbiota composition in response to the reduction or exclusion of gluten from the diet in healthy adults have already been reported. Although these studies have shown some heterogeneous results [1,3,7], the observations reported a decrease in the population level of beneficial bacteria such as *Bifidobacterium* sp. as well as in carbohydrate-utilising species, suggesting that a low or gluten-free diet may shape the microbiota composition and gut health.

The inconsistent outcomes between studies may be partly due to the different gut microbiota sampling and analysis used (sample collection, molecular analyses, region of the 16S rRNA gene studied, etc.) as well as to the small number of interventional studies in healthy individuals. In addition, reducing gluten in the diet means replacing dietary fibre from gluten-rich cereals such as cellulose or hemicellulose and FODMAPs (fermentable oligo-, di-, monosaccharides, and polyols) with dietary fibre from other sources (e.g., maize, sorghum, rice) containing mainly starch or β-glucans. Changes in the gut microbiota may thus be partly due to dietary fibre modification rather than gluten reduction alone, microbial changes being highly dependent on the type of dietary fibre used for replacement [1]. Finally, the heterogeneity of the results could also be due to the different durations of the gluten-free or low-gluten diets studied (from 4 to 8 weeks).

In order to improve our understanding of the effects of a low-gluten diet (LGD) on healthy individuals, we investigated the impact of prolonged exposure to such a diet on the composition and metabolic activity of the gut microbiota of healthy French adults. Healthy men and women who consumed an average of 160 g of bread and pasta per day (i.e., a gluten intake of about 14 to 15 g per day from these foods) were recruited for the study. We compared the gut microbiota structure of these volunteers who switched from their usual gluten-containing diet (HGD, high-gluten diet) to LGD for two successive periods of 8 weeks. LGD was achieved by replacing pasta and bread made with wheat flour with their equivalents made with rice and maize flours (estimated reduction in gluten intake of about 15 g per day). The results were obtained from a randomised controlled trial involving 40 healthy adults without any gluten-related disease. Gut microbiota composition was assessed by 16S rRNA gene sequencing, molecular quantification by PCR, and a cultural approach. The metabolic capacity of the gut microbiota was estimated by measuring faecal fermentative metabolites by ^1^H NMR.

## 2. Material and Methods

### 2.1. Study Design

The intervention study was a single-centre, controlled, randomised trial consisting of two 8-week dietary intervention periods (M2 and M4). After baseline (M0), subjects (*n* = 40) switched to the low-gluten diet (LGD) for an 8-week period (M2) while a group of 20 subjects (*n* = 20) prolonged LGD for another 8-week period (M4). The intervention study was registered at www.clinicaltrials.gov (NCT 03101410), and approved by the Committee for the Protection of Human Subjects (CPP) Sud Est VI, Clermont-Ferrand. All volunteers gave their written informed consent to participate in the study.

Twelve women and twenty-eight men completed the study. Participants were aged between 20 and 50 years (mean 36.1 ± 8.5), have a normal BMI (mean 22.3 ± 1.8), were healthy (reported free of any disease), consumed a usual high-gluten diet (intake of gluten estimated to be 15 g per day on average from wheat-based products such as pasta and bread), and were not receiving any medical treatment. They had no known food intolerances and did not suffer from celiac disease or other gastrointestinal disorders. Exclusion criteria included any medical or surgical history deemed incompatible by the investigator, antibiotic treatment within 2 months before the beginning of the study, and a regular gluten-free diet or any other excluding diet.

The effect of LGD on faecal microbiota composition and metabolism, described in this article, was predefined as a secondary outcome of the study and therefore represented a separate part of the whole clinical trial. The protocol design allowed the collection of 40 faecal samples at baseline (M0) and after 8 weeks of LGD (M2), respectively, and 20 faecal samples after 16 weeks of LGD (M4).

### 2.2. Diet Intervention

The aim of the intervention period was to reduce the daily gluten intake during the LGD period while gluten-containing foods were consumed during the usual diet (HGD). The average daily gluten intake (HDG) was calculated to be approximately 15 g per day from bread and pasta consumption (on average, subjects consumed daily 100 g of bread and 60 g of pasta containing 10% and 7% of gluten, respectively), which is in accordance with the estimation of 10 g to 20 g per day in the European population [8,9]. During the LGD period, participants were given a selection of gluten-free products (pasta and bread), made by replacing wheat flour with rice (50%) and maize flour (50%), and by adding psyllium (quantity not communicated by the supplier) as fibre source to the bread. The participants were instructed to replace all of these cereal-derived products from their usual diet with the gluten-free products provided and to consume these products ad libitum. All products supplied were commercially available and have been provided free of charge to participants. The LGD studied reduced the daily gluten intake by approximately 15 g per day which could be considered a reduction in total gluten intake of almost 80% compared to the HGD.

Participants completed a detailed four-day food diary at the beginning and at the end of 8-week periods and intakes of total energy, macronutrients, and specific food components were assessed. Consumption of study products was monitored using a diary.

### 2.3. Faecal Samples Collection and DNA Extraction

Faecal samples were collected at the beginning and at the end of each diet period and were stored at +4 °C for a maximum of 15 h. They were then aliquoted into cryotubes and immediately frozen at −80 °C and stored for 2 to 3 months until DNA extraction. Total DNA was extracted from these faecal samples using the method described by Godon et al. [10]. Extracted DNA was kept frozen at −20 °C for long-term storage.

### 2.4. 16S Ribosomal RNA (16S rRNA) Gene Sequencing

Bacterial composition from 36 faecal samples collected at M0 (*n* = 12), M2 (*n* = 12) and M4 (*n* = 6) was studied from extracted DNA. The V3–V4 hyper-variable region of the 16S rRNA gene was targeted to analyse bacterial composition. After PCR amplification of the region, paired-end sequencing (2 × 400 bp) was performed using Illumina MiSeq Technology (Life Sequencing, València, Spain). The raw sequences were then trimmed using the QIIME program (Quantitative Insights Into Microbial Ecology), and sequences lower than 200 bp were removed. Taxonomic assignment was based on NCBI database. Clustering of sequence reads into OTUs (Operational Taxonomical Units) at 97% identity level was achieved using QIIME. Clustered sequences processing and statistical microbiota analysis and visualisation were carried out with RStudio 3.5.2 (https://.R-project.org/; accessed on 27 September 2024) (R studio: integrated Development Environment for R. Boston, MA, USA, 2016) with the following packages: phyloseq (v1.40.0) [11], DESeq2 (v1.36.0), rstatix (v0.7.0), microbiomeutilities (v1.00.17), microbiome (v1.18.0), stromboli (v0.1.0.), vegan (v2.6–2), ggplot2 (v3.3.6), ggpubr (v0.4.0). Raw OTU table was filtered and only OTUs present in at least 20% of samples and with 5 reads or more were kept for further analysis. Alpha-diversity was evaluated through total observed OTU, Shannon diversity index and Chao estimates. Difference between timepoints for α-diversity and *Bacteroidota* (formerly *Bacteroidetes*)/*Bacillota* (formerly *Firmicutes*) ratio was evaluated with ANOVA based on linear mixed effects model (LME) followed by pairwise emmeans test with Holm correction and 0.05 significance threshold. Difference in β-diversity (community structures) between the different 8 week-diet periods was calculated using PERMANOVA (adonis function from “vegan” package) with 999 permutations based on Bray–Curtis dissimilarity index. Non-metric multidimensional scaling (NMDS) of distance matrix was used for visualisation. Differences in taxonomic abundance at phylum, family, genus, and OTU level, respectively, between timepoints were assessed using differential abundance analysis based on the negative binomial distribution with Wald test and Benjamini-Hochberg false discovery rate correction with significance threshold of 0.1 and log_2_ fold change threshold of 1.

### 2.5. Quantitative PCR

Population levels of certain phylogenetic groups and bacterial species of interest were quantified in all faecal samples (*n* = 40 at M0, *n* = 40 at M2 and *n* = 20 at M4) by real-time qPCR (Eppendorf Master Cycler ep RealPlex^TM^ 2.0, Hamburg, Germany) through specific amplification of different regions in the 16S rRNA gene. Real-time quantitative PCR was performed using the Brilliant SYBR Green system (Roche, Manheim, Germany) with primers and conditions previously described for *Bacteroides-Prevotella-Porphyromonas* group [12], *Bacillota* (formerly *Firmicute*) [13], *Verrucomicrobiota* [14], *Bifidobacterium* spp. [15], *Lactobacillus-Leuconostoc-Pediococcus* [12], *Faecalibacterium prausnitzii* [16], *Akkermansia muciniphila* [17], and *Escherichia coli* [18].

### 2.6. Cultural Evaluation of Total Anaerobes, Enterobacteriaceae, and the Gluten-Degrading Community Levels

One gram of each freshly collected faecal sample (*n* = 40 at M0, *n* = 40 at M2, and *n* = 20 at M4) was diluted 10-fold (wet *w/v*) in an anaerobic mineral solution. Serial 10-fold dilutions down to 10^−12^ were then made and used to inoculate specific enumeration media.

*Enterobacteriaceae* were enumerated on selective MacConkey agar media (Roth) incubated for 2 days at 37 °C under aerobic conditions. Total anaerobes and the gluten-degrading community were enumerated under strict anaerobic conditions using the Most Probable Number (MPN) method as previously described [19]. All handling (medium preparation, inoculation, dilution …) was performed under 100% CO_2_ gas atmosphere. A series of three liquid culture tubes was inoculated per faecal dilution (10^−3^ to 10^−11^) for each selective medium.

Total anaerobes were enumerated in the Leedle and Hespell medium [20] after 2 days of incubation at 37 °C with bacterial growth as the positive criterion.

The level of the gluten-degrading population was assessed in liquid BC medium [21] with gluten as the sole energy source. A 20 g·L^−1^ solution of wheat gluten (Sigma-Aldrich, Saint-Quentin-Fallavier, France) was obtained in distilled water after passing through an ultrasonic bath, 2 times for 5 min. The final gluten concentration of 1 g·L^−1^ in the culture medium was obtained by adding 200 µL of gluten solution to 8 mL of culture medium. Culture tubes were inoculated with 0.5 mL of faecal dilutions and were incubated at 37 °C for 7 days. The level of the gluten-degrading population was assessed by the MPN method using bacterial growth and gluten degradation as criteria. Gluten utilisation was evaluated by measuring the residual gluten concentration in the culture media using the ELISA method (INgezim Gluten Quick kit, Eurofins Scientific, Nantes, France).

### 2.7. Isolation and Identification of the Main Gluten-Degrading Bacterial Species

The main gluten-degrading bacteria were isolated from faecal cultures (obtained from 10^−6^–10^−8^ faecal dilutions) showing gluten degradation. The medium used to isolate these strains was the solid semi-synthetic BC medium containing 0.5 g·L^−1^ of gluten as the sole energy source. The strains were isolated using the roll-tube method as described previously [19]. After 3 to 5 successive subcultures on roll tubes and broth-gluten BC medium, isolates were examined for purity in gluten-grown cultures by phase contrast microscopy.

Pure gluten-degrading isolates were then identified by 16S rRNA gene sequencing as previously reported [22]. Briefly, cells obtained from gluten BC medium after 24 to 48 h incubation at 37 °C, were harvested by centrifugation for 15 min at 9000× *g* at 4 °C. The bacterial pellet was then subjected to DNA extraction (Easy DNA^TM^ kit Genomic DNA Isolation, Invitrogen BV, Groningen, The Netherlands). The 16S rRNA gene was amplified using the universal primers F8 (5′-AGAGTTTGATCMTGGC-TC-3′) and 1492R (5′-GNTACCTTGTTACGACTT-3′). Approximately 30 ng of purified PCR product was included in a 50 µL sequencing reaction. Sequencing was then performed by Eurofins GATC (Konstanz, Germany). BLAST (version Blast +2.16.0) of the 16s rRNA gene sequences (NCBI database) was used for homology search. A threshold of 99% sequence homology was retained for identification of the strains.

### 2.8. Faecal Fermentative Metabolites Analysis

Bacterial fermentative metabolites were quantified in faecal water of all samples (*n* = 40 at M0, *n* = 40 at M2 and *n* = 20 at M4) by 1D ^1^H NMR using a Bruker Avance III 400 MZ spectrophotometer [23]. Faecal water was obtained from 1 g of each faecal sample kept frozen at −20 °C according to the method described by Zhao et al. [24].

### 2.9. Statistical Analysis

Statistical analyses of qPCR, bacterial metabolites, and cultural enumeration results were performed using the GraphPad InStat 3 software (La Jolla, CA, USA). The statistical test used was the Kruskal–Wallis with Dunn’s multiple comparisons test, except when indicated. Level used to establish significance was *p* < 0.05.

## 3. Results

### 3.1. Food Intake

**Figure 1:**
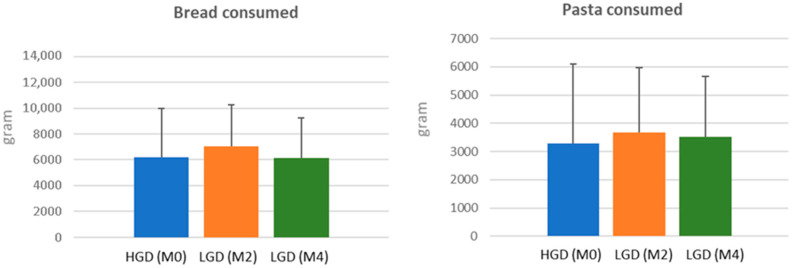
Amounts of bread and pasta consumed (in grams) during usual gluten diet (HGD) (M0) and after low-gluten diet period (LGD) followed for 8 weeks (M2) and 16 weeks (M4).

Analysis of food records from participants showed that the weekly consumption of bread and pasta was similar within the LGD and HGD diets (approximately 800 g of bread and 450 g of pasta) (Figure 1). In HGD, this was equivalent to about 15 g of gluten per day from bread and pasta while no gluten originated from these foods during the LGD. There were no significant differences in dietary macronutrient composition and in energy intake between the two diets (Table 1) except for a slight reduction in protein intake during LGD (14.3% for LGD and 15.9% for HGD).

### 3.2. Evolution of Gut Microbial Diversity and Richness After a Low-Gluten Diet

A total of 1,742,283 16S rRNA reads were obtained from faecal samples of subjects after HGD or LGD, with an average of about 48,500 reads per sample. For each of the samples, a rarefaction curve has been obtained that reached a saturation point, establishing that additional sampling would be of limited benefit.

**Figure 2:**
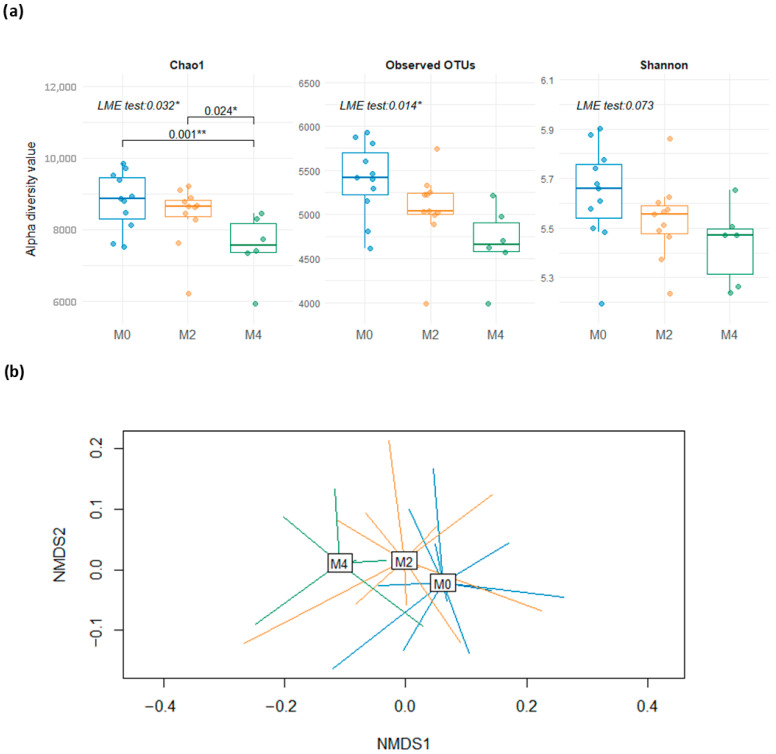
Changes in gut microbiota after 8 (M2) and 16 (M4) weeks on low-gluten diet compared to usual gluten-containing diet at baseline (M0). (**a**) α-diversity at the operational taxonomic unit (OTU) level estimated by Chao1, observed OTUs, and Shannon estimators (* *p* < 0.05; ** 0.0001 < *p* < 0.001); (**b**) non-metric multidimensional scaling (NMDS) based on Bray–Curtis distance, showing differences in diversity between samples (β-diversity) at OTU-level community.

From these data, we first investigated the effect of the LGD consumed for 8 (M2) and 16 weeks (M4), respectively, on the α-diversity of the gut microbial community compared to HGD (M0) using different diversity measures (Observed OTU, Shannon, and Chao1 indexes). The microbial community richness significantly declined during the LGD periods, as measured by Chao1 (*p* = 0.032) and observed OTU (*p* = 0.014) indices, but it was also seemingly proportional to the length of the LGD (Figure 2a).

The variability of the abundance of the microbial community was then analysed among subject groups to investigate potential differences in β-diversity at baseline and after 8 and 16 weeks of LGD, respectively. NMDS, based on Bray–Curtis distance of the 16S rRNA gene sequences, highlighted a clear clustering of microbial populations of the LGD periods (8 and 16 weeks) away from that on the HGD baseline (Figure 2b). The LGD had a clear effect on samples’ β-diversity (PERMANOVA *p* = 0.011), where LGD periods clustered together (M2 vs. M4, *p* = 0.34) while significantly distinct from the HGD baseline (M0 vs. M2, *p* = 0.015, M0 vs. M4, *p* = 0.031).

### 3.3. Variability in Relative Abundance of the Gut Microbial Groups After a Low-Gluten Diet

**Figure 3:**
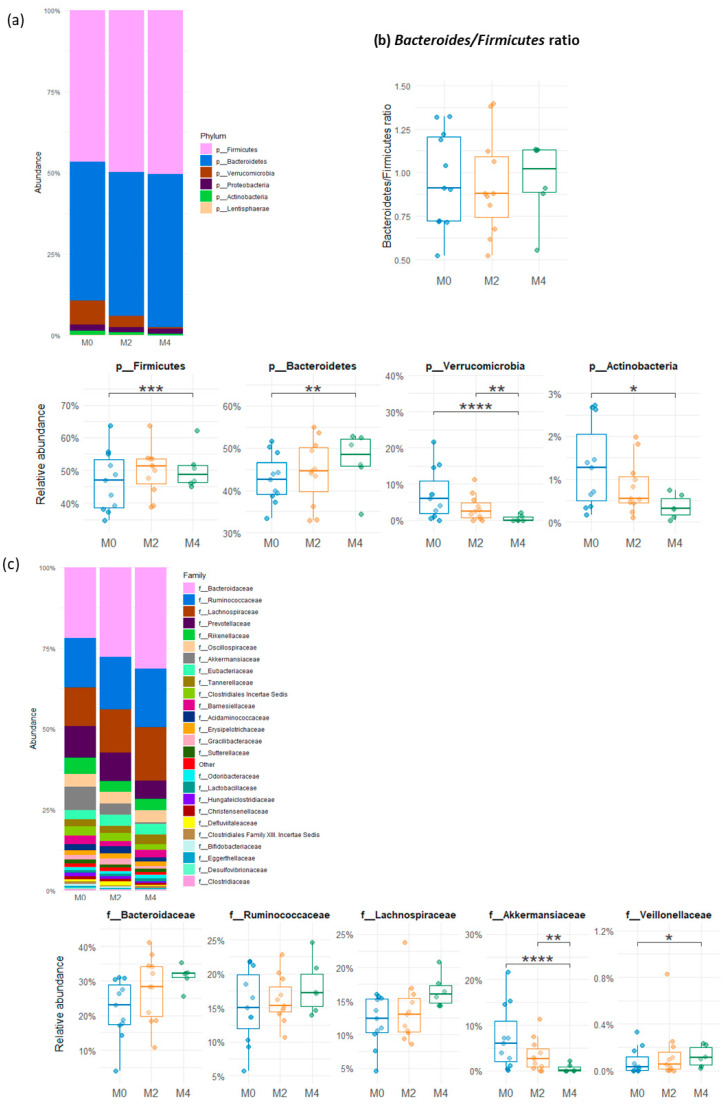
Relative abundance of the bacterial phyla and bacterial families in faecal samples from subjects on usual gluten-containing diet (M0), and after 8 weeks (M2) and 16 weeks (M4) of low-gluten diet. (**a**) Phyla composition of gut microbiota at M0, M2 and M4 and changes in the relative abundance of the predominant phyla (Wald’s test, * *p* < 0.05, ** 0.001 < *p* < 0.01, *** 0.0001 < *p* < 0.001, **** *p* < 0.0001) between baseline (M0) and after 8 weeks (M2) and 16 weeks (M4) of low-gluten diet. (**b**) Box plots showing the *Bacteroidota* (ex *Bacteroidetes*)/*Bacillota* (ex *Firmicutes*) ratio in faecal microbiota of subjects at baseline, on usual gluten-containing diet (M0), and after 8 weeks (M2) and 16 weeks (M4) of low-gluten diet (Anova test). (**c**) Bacterial family composition of the gut microbiota at M0, M2, and M4 and changes in relative abundance of some predominant families (Wald’s test, * *p* < 0.05, ** 0.001 < *p* < 0.01, **** *p* < 0.0001) at baseline (M0) and after 8 weeks (M2) and 16 weeks (M4) of low-gluten diet.

Analysis of the differential abundance based on pairwise comparison between time points (M0 vs. M2, M0 vs. M4, and M2 vs. M4) showed that, at the phylum level, the relative abundance of *Verrucomicrobiota* (formerly *Verrucomicrobia*) and *Actinomycetota* (formerly *Actinobacteria*) was significantly decreased after 16 weeks of LGD compared to baseline (*p* < 0.0001 and *p* = 0.0106, respectively) while *Bacteroidota* (formerly *Bacteroidetes)* and *Bacillota* (formerly *Firmicutes*) were significantly increased (Figure 3a). *Verrucomicrobiota* and *Actinomycetota* were shown to decrease between M2 and M4, although this was only statistically significant for *Verrucomicrobiota*. The ratio of *Bacillota* to *Bacteroidota* did not change between HGD and LGD, whatever the duration of the diet (8 and 16 weeks) (Figure 3b).

At the family level (Figure 3c), the relative abundance of *Akkermansiaceae* was significantly decreased after 16 weeks of LGD (*p* < 0.0001) while *Veillonellaceae* increased in the same period (*p* = 0.05).

**Figure 4:**
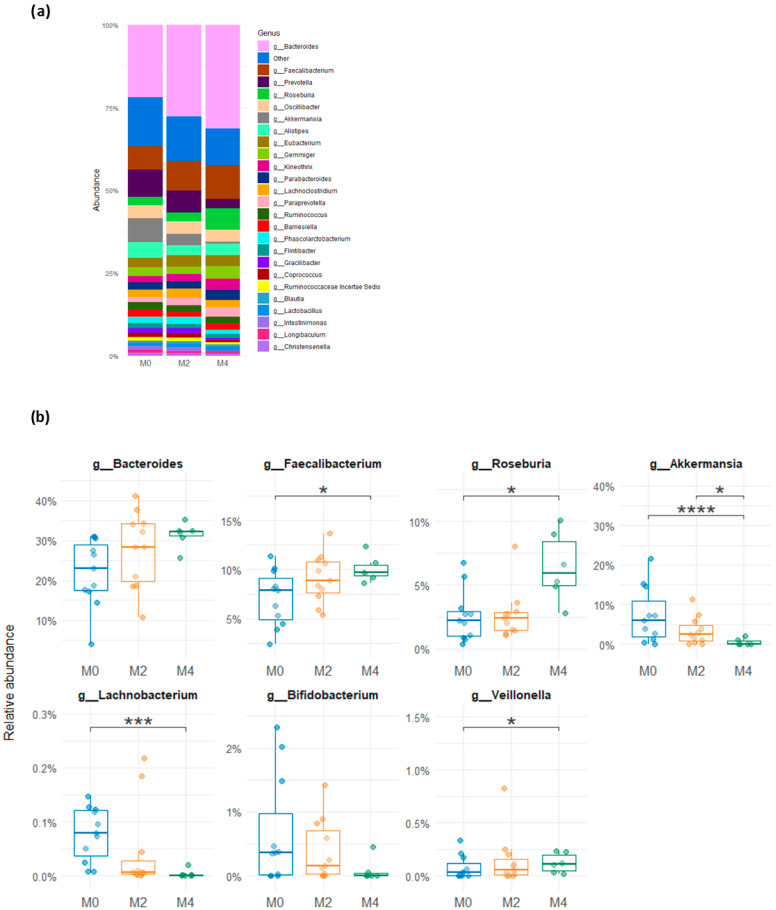
Relative abundance of bacterial genera in faecal samples from subjects on usual gluten-containing diet (M0), and after 8 weeks (M2) and 16 weeks (M4) of low-gluten diet. (**a**) Genera composition of gut microbiota at M0, M2, and M4. (**b**) Change in relative abundance of the predominant genera (Wald’s test, * 0.05 < *p* < 0.1, *** 0.0001 < *p* < 0.001, **** *p* < 0.0001)) between baseline (M0) and after 8 weeks (M2) and 16 weeks (M4) of low-gluten diet.

Among the bacterial genera (Figure 4a,b), LGD induced a significant decrease in the relative abundance of *Akkermansia* (*p* < 0.0001) and *Lachnobacterium* (*p* = 0.0004) after 16 weeks compared to baseline while *Faecalibacterium*, *Roseburia,* and *Veillonella* increased (*p* < 0.1). A decrease in *Bifidobacterium* could also be observed between baseline, M2, and M4 of LGD but failed to be significant because of high inter-individual variation in abundance of this bacterial family.

**Figure 5:**
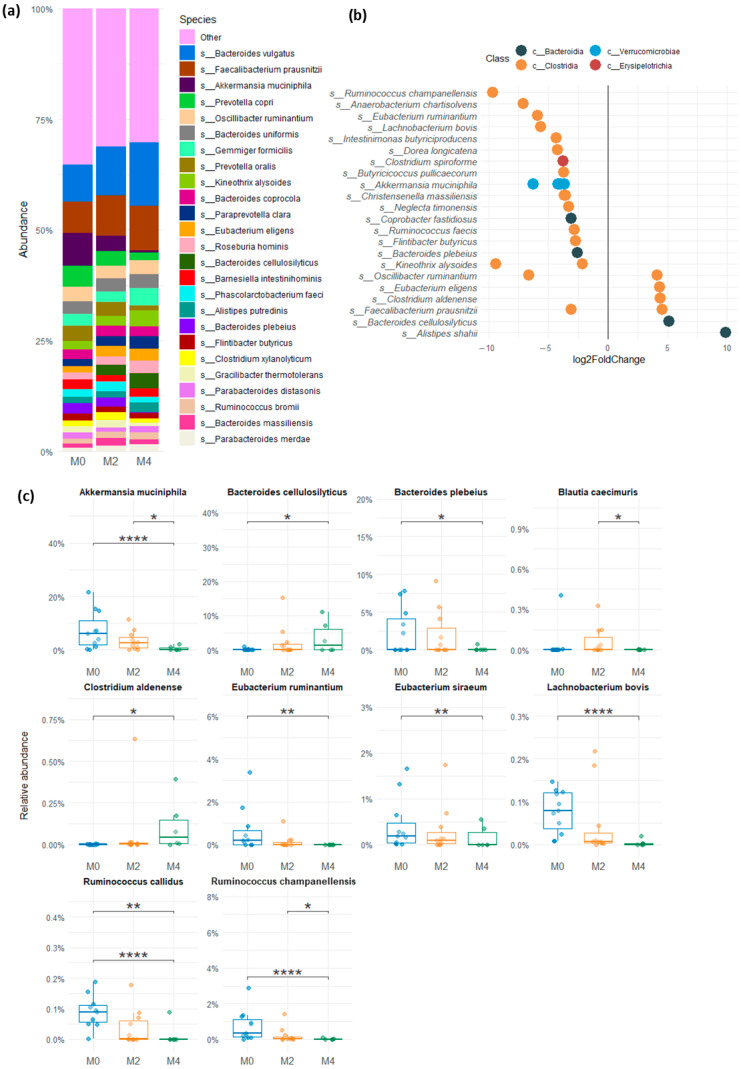
Relative abundance of bacterial species in faecal samples from subjects on usual gluten-containing diet (M0), and after 8 weeks (M2), and 16 weeks (M4) of low-gluten diet. (**a**) Species composition of gut microbiota at M0, M2, and M4. (**b**) Differential species abundance in faecal microbiota between baseline (M0) and after 16 weeks (M4) of low-gluten diet (significant annotated species that showed a Log 2 fold change in relative abundance between baseline and 16 weeks (M4) of low-gluten diet—adjusted *p*-value < 0,05). (**c**) Change in relative abundance of the predominant annotated species (Wald’s test, * *p* < 0.05, ** 0.001 < *p* < 0.01, **** *p* < 0.0001)) between baseline (M0) and after 8 weeks (M2) and 16 weeks (M4) of low-gluten diet.

At the species level (Figure 5), LGD resulted in significant differential abundance of several species from the *Bacteroidia*, *Verrucomicrobiae*, and *Clostridia* classes, compared to baseline. Among these annotated species, *Akkermansia muciniphila*, one of the main mucin-degrading species, significantly decreased after 16 weeks of LGD (*p* < 0.0001) as well as between 8 and 16 weeks of LGD (*p* = 0.0473) (Figure 5b,c), corroborating the observed decrease in the genus *Akkermansia*. Similarly, the significant decrease in *Lachnobacterium bovis* (*p* < 0.0001), a lactate-producing bacterium, after 16 weeks of LGD compared to baseline, supports the observed decrease in the *Lachnobacterium* genus. Many fibre-degrading species were also shown to be significantly affected by 16 weeks of LGD (Figure 5c), such as *Ruminococcus champanellensis*, *R. callidus,* as well as *Bacteroides plebeius,* whereas *B. cellulosilyticus* increased. Among the *Lachnospiraceae* family, which includes many butyrate-producing species, *Eubacterium* sp. decreased after 16 weeks of LGD, as well as *Blautia caecimuris*.

### 3.4. Molecular Quantification of Microbial Groups from the Human Gut After a Low-Gluten Diet

**Figure 6:**
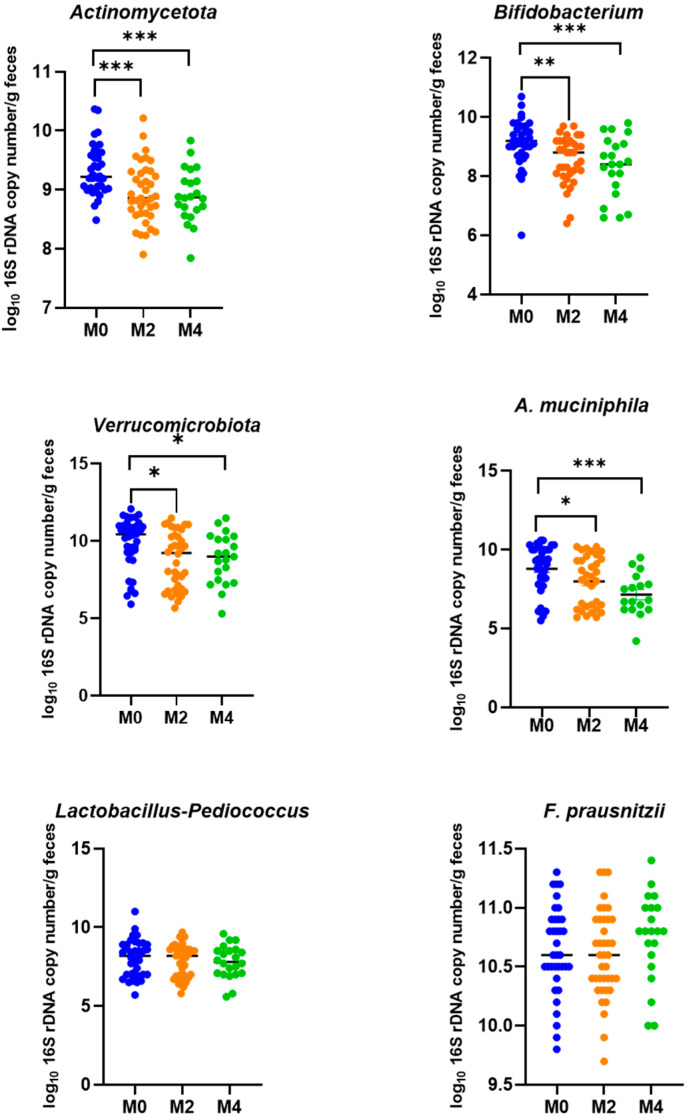
PCR quantification of some bacterial taxa. Main phyla, bacterial groups, and some genera and species of interest were quantified by qPCR using specific primers. Total DNA extracted from faeces of subjects collected at baseline (M0; *n* = 40) on usual gluten-containing diet and after 8 weeks (M2; *n* = 40) and 16 weeks (M4; *n* = 20) on low-gluten diet. Results are expressed as log_10_ copies of 16S rRNA gene g^−1^ faeces (mean ± SD). Comparisons of microbial population level between M0, M2 and M4 were made using the Kruskal–Wallis test (* *p* < 0.05; ** 0.0001 < *p* < 0.001; *** *p* < 0.0001).

QPCR quantification of the major gut bacterial phyla (Figure 6) showed a significant decrease in *Actinomycetota* (*p* = 0.0003, One-way ANOVA) and in *Verrucomicrobiota* (*p =* 0.0213, One-way ANOVA) after LGD periods compared to baseline while no changes were observed for the *Bacteroidota* and *Bacillota* phyla. The reduction in *Actinomycetota* is supported by the significant decrease in the genus *Bifidobacterium* (*p* = 0.0021, One-way ANOVA) after the two LGD periods. The decrease in *Verrucomicrobiota* population is supported by the significant decrease in the species *Akkermansia muciniphila* after LGD periods (*p* = 0.001, One-way ANOVA) compared to baseline, confirming also results obtained by 16S rRNA gene sequencing. No changes were detected in the *Lactobacillus*-*Pediococcus* group, as well as in the bacterial species *Escherichia coli* and *Faecalibacterium prausnitzii*.

### 3.5. Evolution of Cultivable Total Anaerobes, Enterobacteriaceae and Gluten-Degrading Community

**Figure 7:**
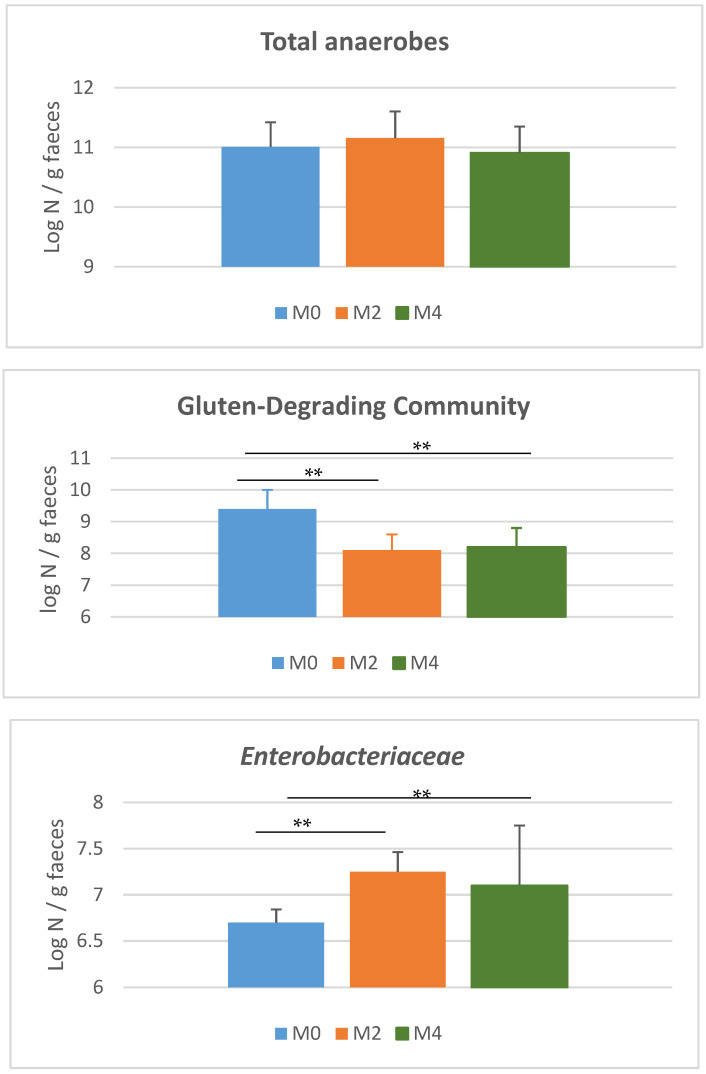
Distribution of culturable bacterial communities (total anaerobes, gluten-degrading community and *Enterobacteriaceae*) in faecal samples from subjects at baseline (M0; *n* = 40) on a usual gluten-containing diet and after 8 weeks (M2; *n* = 40) and 16 weeks (M4; *n* = 20) of low-gluten diet. Comparisons of the population levels of microbial communities (expressed as log_10_ microorganisms per gram of faeces) between M0, M2, and M4 were made using the Kruskal–Wallis test (** 0.0001 < *p* < 0.001).

The population level of total anaerobes remained similar to baseline (M0) regardless of the duration of the LGD. In contrast, the *Enterobacteriaceae* population increased 10-fold after 8 and 16 weeks of LGD (Figure 7).

The gluten-degrading community level was assessed by culture method using a specific culture medium containing gluten (1 g·L^−1^) as the sole energy source (Figure 7). This approach allowed quantification of this community at a baseline level (under a gluten-containing diet: HGD) of approximately 10^9^ bacteria per gram of faeces. After 8 weeks of LGD, the level of the gluten-degrading community decreased significantly by about 10-fold (*p* = 0.0001), stabilising and showing no further decrease after an additional 8 weeks of LGD.

### 3.6. Analysis of Faecal Fermentative Metabolites

**Figure 8:**
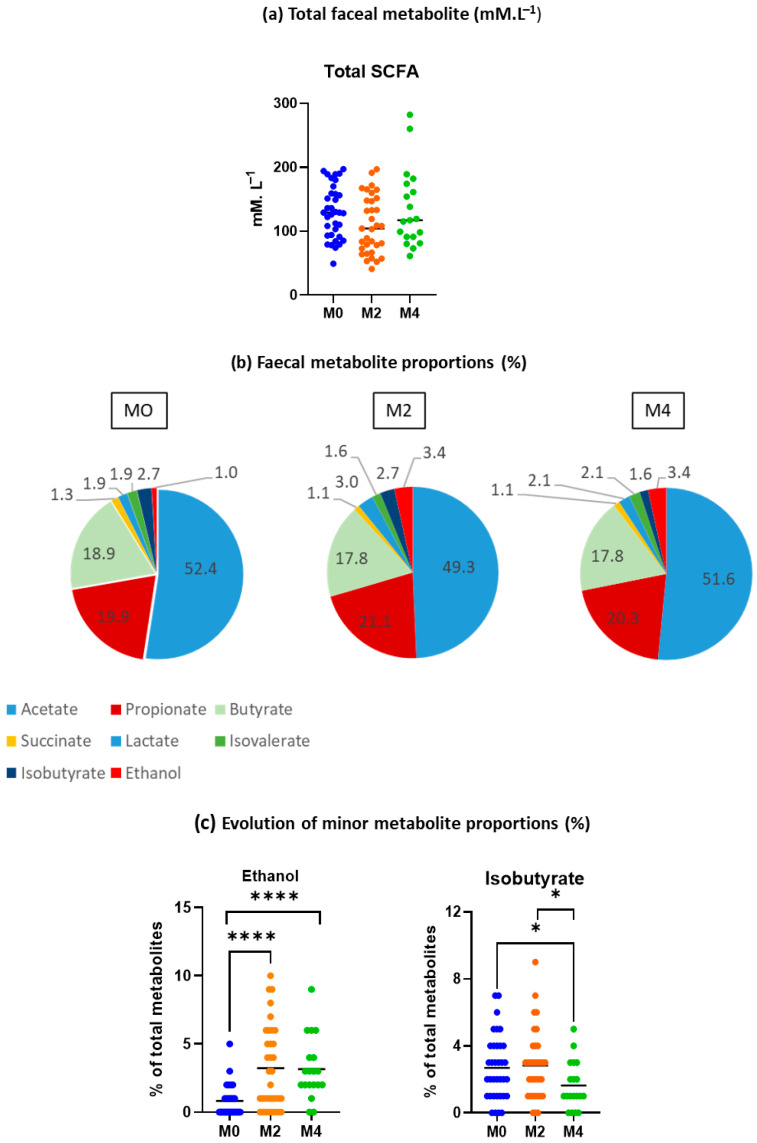
Faecal profile of fermentative metabolites before (M0; *n* = 40) and after 8 weeks (M2; *n* = 40) and 16 weeks (M4; *n* = 20) of low-gluten diet. (**a**) concentrations of total metabolites expressed in mmol·L^−1^ (**b**) Relative proportions of fermentative metabolites detected (expressed as percentages of total metabolites) (**c**) Evolution of isobutyrate and ethanol (expressed as percentage of total metabolites) after 8 and 16 weeks of LGD. Data are presented as mean ± SD and comparisons were made using the Kruskal–Wallis test (* *p* < 0.05, **** *p* < 0.0001).

Total concentration of faecal fermentative metabolites was not significantly different after 8 and 16 weeks of LGD (Figure 8a). After 8 weeks of LGD, the proportion of acetate was slightly reduced compared to baseline (*p* = 0.0347), in favour of propionate (Figure 8b), as also shown by the analysis of the ratio between the three main short-chain fatty acids (acetate/propionate/butyrate: 58/22/20 vs. 56/24/20 vs. 57/23/20 at M0, M2, and M4, respectively). Minor fermentative metabolites (succinate, lactate, isovalerate, isobutyrate, and ethanol) seemed to be more affected by the LGD (Figure 8c). Ethanol was particularly concerned: its proportion was significantly increased (by more than 3 times) after 8 and 16 weeks of LGD (*p* < 0.0001) (Figure 8c). On the contrary, isobutyrate was significantly decreased after 16 weeks of LGD (M4) but not after 8 weeks of diet (M2).

### 3.7. Composition of the Cultivable Gluten-Degrading Community

The main gluten-degrading strains were isolated from the gluten-enriched cultures obtained from faecal samples of 6 volunteers at baseline (M0), while adhering to HGD. Pure isolates, able to grow on BC medium with gluten (1 g·L^−1^) as the sole energy source, were successfully obtained, and 12 of them, representative of the collection and of each donor, were identified through sequencing of their respective 16S rRNA gene, followed by BLAST analysis (Table 2). Most of the isolates belong to the class of *Clostridia*, three to *Erysipelotricha*, two to *Gammaproteobacteria*, and one isolate to *Actinomycetota*. Among the *Clostridia*, 5 strains belong to the *Lachnospiraceae* family and correspond to four different species: *Enterocloster boltae* (found in two subjects), *Enterocloster clostriodiformis* (one subject), *Enterocloster citronae* (one subject), and *Hungatella hathewayi* (one subject). An isolate from the family *Oscillospiraceae* was identified as *Flavonifractor plautii*. The *Erysipelotricha* strains belonging to the *Erysipelotrichaceae* family were found in 3 subjects: the isolate from subject 1 showed 100% 16S rRNA gene sequence homology with *Thomasclavelia ramosa* (formerly *Clostridium ramosum*) while the two other isolates, from subjects 1 and 4, were identified as *Clostridium innocuum*. The two *Gammaproteobacteria* isolated from two subjects correspond to *Enterobacteriaceae* with 100% 16S rRNA gene sequence homology with *E. coli*. One subject was shown to harbour an *Actinomycetota* strain as the main gluten-degrading bacteria, with the representative isolate corresponding to *Bifidobacterium longum* (100% 16S rRNA gene sequence homology).

## 4. Discussion

In this study, we showed that a low-gluten diet for several months induced major changes in the composition of the gut microbiota of apparently healthy French adults. Microbial changes were already observed after 8 weeks of a low-gluten diet as previously reported in other European populations [1,3,7], but they were more moderate than those observed after 16 weeks of LGD.

The low-gluten diet was mainly associated with a decrease in microbial richness, especially in the longer duration period. Gut microbiota richness is considered an important parameter in the host-microbiota symbiosis. In this respect, low gut microbiota richness has been observed in a wide range of pathologies [25,26] but other parameters such as diet may also influence gut microbiota richness, independent of health status [25].

At the taxonomic level, 16S rRNA gene sequencing results clearly showed that a low-gluten diet significantly affected the abundance of several taxa. The dominant phyla *Bacteroidota* and *Bacillota* were similarly affected by the low-gluten diet, so that the *Bacillota*/*Bacteroidota* ratio was not different before and after the diet. At the phylum level, one of the most important changes observed after a low-gluten diet was the drastic reduction in *Verrucomicrobiota,* along with significantly reduced abundance of the *Akkermansiaceae* family, the *Akkermansia* genus, and the *Akkermansia muciniphila* species. Both the phylum and species levels, as quantified by the qPCR approach, further sustained this finding. To our knowledge, this is the first report on the effect of a low-gluten diet on *Akkermansia muciniphila*. *A. muciniphila* is a mucin-degrading bacterium representing 1 to 4% of the faecal microbiota in healthy individuals [27]. The contribution of *A. muciniphila* to host-microbiota homeostasis has been largely reported [28,29]. Indeed, this bacterial species is recognised as a key player in the maintenance of intestinal barrier integrity and regulation of the immune response through limiting the inflammation onset, the underlying cause of various diseases. Low levels of *A. muciniphila* have been found in several pathologies, particularly obesity and obesity-related disorders such as type 2 diabetes and non-alcoholic fatty liver disease. When administered to obese human volunteers, *A. muciniphila* was shown to improve metabolic parameters such as insulin resistance, demonstrating its crucial role in glucose regulation [30]. As a higher risk of metabolic syndrome has been reported in celiac disease patients one year after starting a gluten-free diet [6], it would be interesting to determine whether the relative abundance of *A. muciniphila* is also affected in these patients. How a low- or gluten-free diet might affect the abundance of *A. muciniphila* remains however to be investigated. One hypothesis is that the reduction in *A. muciniphila* abundance is due to changes in dietary fibre content in the low-gluten diet, particularly the fermentable oligo-, di, monosaccharides, and polyols (FODMAPs) commonly found in wheat. Indeed, oligofructose administration has been reported to restore the *A. muciniphila* population in diet-induced obese mice [27], suggesting that reducing the amount of FODMAPs may induce a reduction in the *A. muciniphila* population. Another hypothesis is related to the reduced polyphenol content that characterises gluten-free products compared to gluten-containing products [31]. Polyphenols are important micronutrients that play a key role in human health by strengthening the intestinal mucosal barrier through increased mucin production and modulation of gut microbiota. The reduced levels of polyphenols in low- or gluten-free diets may therefore weaken the mucus barrier [32,33] and consequently affect mucin-degrading species such as *A. muciniphila*. The exact effect of a low- or gluten-free diet on the *A. muciniphila* population therefore requires further investigation.

The other phylum that appeared to be significantly reduced after 16 weeks of the low-gluten diet was *Actinomycetota*. In this phylum, *Bifidobacterium* was the bacterial genus that was most affected by the low-gluten diet. qPCR quantification clearly showed a significant decrease in this genus after both 8 and 16 weeks of the diet, as previously observed [1,7], while analysis of the relative abundance of *Bifidobacterium* showed a similar profile, although not significant. A lower abundance of *Bifidobacterium* has also been reported in celiac disease patients on a gluten-free diet [34]. It has been suggested that the reduced abundance of *Bifidobacterium* observed in a low-gluten diet is mainly due to the reduction in dietary FODMAPs [1]. *Bifidobacterium* species can indeed grow on many of the wheat FODMAPs, and a low-FODMAP diet has been shown to affect the abundance of *Bifidobacteria* in irritable bowel syndrome patients [35,36]. However, it has also been reported that the gluten-degrading community of the gut microbiota includes *Bifidobacterium* strains [37]. Similarly, we have isolated a strain of *Bifidobacterium longum* that is able to grow on gluten as the sole substrate. This suggests that the reduced amount of gluten may directly affect the abundance of *Bifidobacteria* in healthy adults.

We also observed a decrease in the abundance of some fibre-degrading species belonging to *Ruminococcus* sp. (*R. champanellensis*, *R. callidus*). In contrast, fibre-degrading *Bacteroides* such as *Bacteroides cellulosilyticus*, were increased. *Ruminococcus* sp. are more specialised than *Bacteroides* sp. in the degradation of polysaccharides from plant cell walls [19,21], which probably explains why they were more affected by the depletion of such fibres associated with the low-gluten diet than *Bacteroides* sp., which are able to use a larger variety of polysaccharides. A low-gluten diet for a longer period was also shown to differentially affect butyrate-producing bacteria. A decrease in the abundance of some *Lachnospiraceae* species such as *Lachnobacterium* sp., *Eubacterium* sp. (*E. ruminantium*, *E. siraeum*) and some *Clostridia* species (*Intestinimonas butyriciproducens* or *Flintibacter butyricus*) was observed. But other *Lachnospiraceae* butyrate-producing species such as *Roseburia* or other *Eubacterium* (*E. eligens*) as well as *Faecalibacterium*, and some *Clostridium (C. aldenense)* were increased after a low-gluten diet. Changes in the structure of microbial communities that degrade fibres and/or produce butyrate may be due to the different types and quantities of fibre contained in wheat flour compared to the enriched-starch rice and corn flours which are used in gluten-free foods. Finally, although the low-gluten diet modulated the diversity of the butyrate-producing community, there was no effect of this microbial change on total butyrate production, due to the redundant butyrate-synthesising capacity of many different bacterial taxa.

Many gluten-free foods are ultra-processed industrial formulations containing additives such as emulsifiers, stabilisers, and sweeteners to improve sensory scores and extend shelf life. Emulsifiers, which are detergent-like molecules, are ubiquitous components of processed foods that may disrupt mucus-bacterial interactions and have the potential to promote diseases associated with gut inflammation. Chassaing et al. [38] demonstrated that the administration of two emulsifiers to a mouse model caused alterations in the composition of the gut microbiota, including a reduction in microbial diversity and increased levels of several mucin-degrading species, such as *Ruminococcus gnavus* and *Akkermansia muciniphila*, as well as an enrichment in mucosa-associated, inflammation-promoting *Proteobacteria*. Therefore, it could be hypothesised that some of the alterations to the gut microbiota observed in healthy subjects on a low-gluten diet may be due to an increased intake of food additives in the gluten-free products supplied.

Finally, culture counts of *Enterobacteriaceae* showed a significant increase in this bacterial population after 8 and 16 weeks of a low-gluten diet, as previously reported [3,7], although we did not observe such an effect using molecular approaches. Thus, the effect of the low-gluten diet on this bacterial population (and especially on *E. coli*) remains unclear and requires further investigation.

Despite the microbial changes induced by the low-gluten diet, we did not observe major effects on the production of main bacterial fermentative metabolites (i.e., acetate, propionate, and butyrate) as also previously reported [1]. A significant increase in the production of ethanol was nevertheless observed following the low-gluten diet periods. Ethanol is produced from the fermentation of sugars by a variety of bacteria present in the gut such as *Limolactobacillus* sp., *Alistipes communis*, *Mediterraneibacter* (formerly *Ruminococcus*) *gnavus* [39], or *E. coli*, but its concentration remains usually very low. Since ethanol recycling by gut microorganisms has never been reported, the increase in ethanol concentration after a low-gluten diet may thus be due to an increase in the population level and/or activity of ethanol-producing bacterial species that remain to be identified. In this context, culturomics, metagenomics, and metabolomics are complementary approaches that will be essential in identifying these ethanol-producing bacteria. Increased faecal ethanol has been reported in patients with non-alcoholic steatohepatitis (a disease associated with metabolic syndrome) [39,40], alongside gut microbiota dysbiosis characterised by a high level of *E. coli*, an ethanol-producing bacterium. It has also been reported that insulin resistance causes abnormal ethanol metabolism [40]. Excessive ethanol can cause intracellular redox potential changes that lead to increased inflammatory responses. Particular attention is thus warranted by the overproduction of ethanol linked to a low-gluten diet.

We also observed a significant reduction in isobutyrate production after 16 weeks of a low-gluten diet. Isobutyrate is mainly produced by protein catabolism. Although small, the reduction in protein intake observed after a low-gluten diet could explain the decrease in microbial protein metabolism.

Finally, we characterised the predominant bacterial species of the gluten-degrading community in healthy adults. Most of the isolated strains able to grow on gluten as the sole carbon and nitrogen source, belong to the phylum *Bacillota*, with 65% of the strains belonging to the class *Clostridia*, mostly from the *Lachnospiraceae* family, and to the clade *Clostridium* reclassified as *Enterocloster* [41]. Gluten-degrading activity has previously been found mainly in *Clostridium* species [37], including some species from the family *Lachnospiraceae* that were not further identified [41]. The identification of a strain from the family *Oscillospiraceae* corresponding to *Flavonifractor plautii* and a strain from the family *Coprobacillaceae* (*T. ramosa*) is the first demonstration of gluten-degrading ability in these bacterial species. Gluten-degrading isolates include *E. coli* and *Bifidobacterium longum*, confirming the ability of these taxa to utilise gluten [37,42]. Overall, our results provide further evidence that the ability to utilise gluten is carried by a wide variety of bacterial species. We have also shown that there are inter-individual differences in the composition of the gluten-degrading community, with each individual harbouring its own gluten-degrading bacteria, as previously suggested [42]. Although a low- or gluten-free diet may affect different bacterial taxa depending on the individual, the overall activity of the gluten-degrading community was similarly decreased in all subjects under a low-gluten diet. Ultimately, the isolation of gluten-degrading bacterial species that have a potential health-promoting effect (i.e., *Bifidobacterium* sp.) could enable the development of therapeutic strategies to alleviate gluten-related disorders in people with non-celiac gluten/wheat sensitivity. Administering these gluten-degrading bacteria could improve digestive comfort and reduce sensitivity in such patients by enabling them to digest gluten.

## 5. Conclusions

In conclusion, we showed that a low-gluten diet for several months induced adverse changes in the composition of the gut microbiota of apparently healthy French adults and that prolonged consumption of such a diet leads to deterioration, rather than stabilisation of the gut microbiota in healthy individuals. A low-gluten diet negatively affects the gut microbiota by inducing a reduction in microbial richness and decreasing the abundance of bacterial species with previously reported potential health benefits, such as *Akkermansia muciniphila* and *Bifidobacterium* sp. The change in fibre content, due to the replacement of wheat by rice or maize, also affects the level and diversity of the fibre-degrading community and the butyrate-producing population. The main metabolic effect of these microbial changes was the promotion of ethanol synthesis. This finding requires further investigation, as an increase in this metabolite is likely to be detrimental to host health. Finally, a prolonged low-gluten diet altered the gut microbiota of healthy individuals, shifting it towards a profile that has been associated with adverse health effects. While healthy individuals primarily adopt a low- or gluten-free diet to enhance well-being and digestive comfort, they should be aware of the potential health risks associated with this dietary choice. Evaluating the low-gluten diet over a longer period of time would consolidate these results and enable a more in-depth examination of its impact on the host’s physiology, immunity, and metabolism.

## Figures and Tables

**Table 1:** Energy (Kcal) and protein, carbohydrate, and fat intake during usual gluten diet (HGD) and low-gluten diet (LGD) expressed in grams (g) and in % of total energy intake (ns = no significant difference; *p* > 0.05).

Nutrient	HGD (*n* = 40)		LGD (*n* = 40)		*p*
	Mean	SD	Mean	SD	
Energy (Kcal)	2505.1	561.0	2529.2	600.5	ns
Protein (g)	100.6	22.6	89.6	25.4	ns
Protein (%)	15.9	1.9	14.3	2.2	ns
Carbohydrates (g)	277.2	70.2	272.0	83.7	ns
Carbohydrates (%)	43.2	4.1	42.7	6.5	ns
Fat (g)	101.3	24.3	102.9	26.6	ns
Fat (%)	35.9	4.7	36.8	5.7	ns

**Table 2:** Identification of gluten-degrading strains isolated from faecal samples of 6 subjects on usual gluten-containing diet (HGD) at M0.

Strains	Subject	Identification		
		Class	Family	Species
LG1,5	1	*Clostridia*	*Lachnospiraceae*	*Enterocloster boltae*
JB51	3	*Clostridia*	*Lachnospiraceae*	*Enterocloster boltae*
LG3,1	1	*Clostridia*	*Lachnospiraceae*	*Enterocloster clostridioformis*
M61	6	*Clostridia*	*Lachnospiraceae*	*Enterocloster citronae*
LG4	1	*Clostridia*	*Lachnospiraceae*	*Hungatella hathewayi*
M51	5	*Clostridia*	*Oscillospiraceae*	*Flavonifractor plautii*
LG2,1	1	*Erysipelotricha*	*Erysipelotrichaceae*	*Clostridium innocuum*
L32	4	*Erysipelotricha*	*Erysipelotrichaceae*	*Clostridium innocuum*
LG2,2	1	*Erysipelotricha*	*Coprobacillaceae*	*Thomasclavelia ramosa*
B9	2	*Actinomycetes*	*Bifidobacteriaceae*	*Bifidobacterium longum*
LG1	1	*Gammaproteobacteria*	*Enterobacteriaceae*	*Escherichia coli*
N61	6	*Gammaproteobacteria*	*Enterobacteriaceae*	*Escherichia coli*

## Data Availability

The data underlying this article cannot be shared directly as they are under legislation for data protection. They must be requested from the respective holders after approval of the French Committee for the Protection of Human Subjects Authority.
